# The phenomenon of strain degeneration in biotechnologically relevant fungi

**DOI:** 10.1007/s00253-023-12615-z

**Published:** 2023-06-21

**Authors:** Caroline Danner, Robert L. Mach, Astrid R. Mach-Aigner

**Affiliations:** 1 Christian Doppler Laboratory for Optimized Expression of Carbohydrate-Active Enzymes, Institute of Chemical, Environmental and Bioscience Engineering, TU Wien, Gumpendorfer Str. 1a, 1060 Vienna, Austria; 2 Institute of Chemical, Environmental and Bioscience Engineering, TU Wien, Gumpendorfer Str. 1a, 1060 Vienna, Austria

**Keywords:** Fungi, Strain stability, Degeneration, Biotechnology

## Abstract

**Abstract:**

Fungi are widely exploited for large-scale production in the biotechnological industry to produce a diverse range of substances due to their versatility and relative ease of growing on various substrates. The occurrence of a phenomenon—the so-called fungal strain degeneration—leads to the spontaneous loss or decline of production capacity and results in an economic loss on a tremendous scale. Some of the most commonly applied genera of fungi in the biotechnical industry, such as *Aspergillus*, *Trichoderma*, and *Penicillium*, are threatened by this phenomenon. Although fungal degeneration has been known for almost a century, the phenomenon and its underlying mechanisms still need to be understood. The proposed mechanisms causing fungi to degenerate can be of genetic or epigenetic origin. Other factors, such as culture conditions, stress, or aging, were also reported to have an influence. This mini-review addresses the topic of fungal degeneration by describing examples of productivity losses in biotechnical processes using *Aspergillus niger*, *Aspergillus oryzae*, *Trichoderma reesei*, and *Penicillium chrysogenum*. Further, potential reasons, circumvention, and prevention methods are discussed. This is the first mini-review which provides a comprehensive overview on this phenomenon in biotechnologically used fungi, and it also includes a collection of strategies that can be useful to minimize economic losses which can arise from strain degeneration.

**Key points:**

*• Spontaneous loss of productivity is evident in many fungi used in biotechnology.*

*• The properties and mechanisms underlying this phenomenon are very versatile.*

*• Only studying these underlying mechanisms enables the design of a tailored solution.*

## Introduction

Fungi are a relatively understudied, incredibly versatile group of organisms with enormous value and potential for the biotechnical industry. Due to their adaptation and survival strategies for many habitats, fungi naturally provide many attractive attributes for biotechnological application (Hyde et al. 2019). Hyper-producing strains and heterologous protein expression were enabled by undirected mutagenesis followed by screening and, more recently, by metabolic engineering and genome editing efforts (Hamer 2004). The exploitation of fungi reaches from their nutritional qualities or potential for bioremediation to the production of alcoholic beverages, colorants, biofuels, pharmaceuticals, enzymes, and organic acids with various applications (Adrio and Demain 2003; Hyde et al. 2019). Although production processed using fungi is promising, a common phenomenon, the so-called “fungal degeneration,” challenges long-term cultivation and limits production yields (Song et al. 2022).

The term “degeneration” was first defined by Reusser in 1963 as the following: “culture stability and degeneration refer to the ability of a given microbial population to retain desirable morphological or biosynthetic characteristics qualitatively and quantitatively from generation to generation” (Reusser 1963). From this time point on, several other terms, such as phenotypic instability, phenotypic deterioration, saltation, dual phenomena, phenotypic switch, colony deterioration, or attenuation, emerged that all correspond to this basic definition (Butt et al. 2007; Kawakami 1960; Nagaich 1973, Li et al. 2014, Ibrahim et al. 2002, Ryan et al. 2002). It is notorious that, in an artificial growth environment (for example, growth media, temperature, pH, or nutrient concentrations different to the ones in the natural habitat), many fungal vegetative structures differ phenotypically from their natural form (Stevens 1922; Kuthan et al. 2003). The ability to switch between different phenotypes also occurs in nature and serves as an advantage for fungi to adapt to changing environments (Caten 1994, Friedman et al. 2014; Jain et al. 2008). However, fungal degeneration can also severely affect a whole ecosystem (Khani et al. 2017; Bahram and Netherway 2022). This phenomenon can even affect human health by the development of diseases and infections or pathogenic fungi able to cope with protective measures (Jain et al. 2008; Lohse and Johnson 2008; Moralez et al. 2016; Pietrella et al. 2003; Soll 2009). Finally, degeneration causes difficulties in strain maintenance and productivity and thereby severely affects the biotechnology industry since phenotypic stability is indispensable in an industrial setting (Silar 2019). Consequently, understanding this phenomenon is highly important.

The term fungal degeneration occurs for several different phenomena in literature. Some of these phenomena are shortly introduced in the following section in order to differentiate the term for the rest of the review. Certain basidiomycetes are reported to lose their ability to produce fruiting bodies during cultivation (Magae et al. 2005; Piscitelli et al. 2005; Lee et al. 2014; Chen et al. 2019; Zhu et al. 2020; Pérez et al. 2021; Zhao et al. 2022). *Cordyceps militaris*, for example, is commercially cultivated as a pharmaceutical and food resource and frequently degenerates through subculture and storage. Next to the degeneration of fruiting body formation, secondary metabolite production is reduced, leading to high economic losses (Xiong et al. 2013; Sun et al. 2017; Yin et al. 2017; Lou et al. 2019; Wellham et al. 2021). Their ability to sexually reproduce adds another layer of complexity, which might be the reason why this is not reviewed in the literature. Another interesting phenomenon is the loss of productivity of a secondary metabolite, such as taxol or camptothecin, after storage or sub-cultivation in the lab. This was related to a lack of host stimulus, and productivity could be restored by mimicking environmental stimulations (Li et al. 1998; El-Sayed et al. 2019, 2022). To date, the most thoroughly studied phenotypes of fungal degeneration are related to the loss or attenuation of virulence and different morphological changes of thalli, such as the wooly degeneration, crippled growth, incolore, or senescence phenomenon. For a review of these types of degeneration, it is referred to Butt et al. (2007) and Silar (2019).

This is the first mini-review that specifically focuses on the loss or decline of production capacity of already well-established fungal production strains in the biotechnological industry. Therefore, the term “degeneration” from here on is referred to the changes caused by prolonged cultivation in a laboratory or industrial environment. The production losses in fungal bioprocesses due to this phenomenon are a main issue from an economic perspective. Surprisingly, this topic remained relatively unexplored so far and has not yet been reviewed in the literature. This mini-review is aimed at addressing this gap of knowledge by describing and comparing examples of productivity losses of *Aspergillus niger*, *Aspergillus oryzae*, *Trichoderma reesei*, and *Penicillium chrysogenum* in a biotechnical process. Potential underlying reasons for fungal degeneration, including genetic or epigenetic mechanisms, in addition to external factors relevant to a bioprocess such as culture conditions or stress, are discussed. The review further explores circumvention and prevention methods for fungal strain degeneration. The novelty of this mini-review resides in the detailed description of reported cases leading to productivity losses in the biotechnological industry and includes suggestion for strategies to alleviate the economic loss associated with this phenomenon. Most of the article refers to filamentous fungi; however, in the section about epigenetic mechanisms, it also refers to yeasts because they are the most studied in this research field.

## Degeneration of biotechnologically relevant fungi

The lack of stability of a microbial strain is considered a central challenge in large-scale bioprocesses, ultimately limiting the current absolute necessity to transition to a greener society. Moreover, the implementation of continuous fermentations, despite higher volumetric production capacity, is hampered by the appearance of non-producer cells (Rugbjerg et al. 2018). Studies on the loss or reduction of production capacity in a biotechnical process are rare. Degeneration of culture stability on artificial media is a notorious trait of filamentous fungi (Li et al. 2014; Song et al. 2022). Evidence of degeneration of productivity in large-scale production processes was primarily found in filamentous fungi, including the most commonly applied genera, *Aspergillus*, *Trichoderma*, and *Penicillium*. A summary of the phenotypes of the degeneration phenomenon in common biotechnologically relevant fungi is provided in Table 1.

**Table 1:** The degeneration phenomenon in biotechnologically relevant fungi

Species	Unstable properties	Drop in production	Morphological change	References
*Aspergillus niger*	Citric acid and total organic acid production	30–70%	Smoother spore surface with bigger holes and a thinner, partially lost melanin layer	Xie et al. 2018; Hui et al. 2017
*Aspergillus oryzae*	Hydrolytic enzyme and flavor precursor production	Not quantified	Sectors with white hyphae and sclerotia formation, resulting from abnormal branching and intertwining mycelium	Jin et al. 2011; Zhong et al. 2018
*Penicillium chrysogenum*	Penicillin production	tenfold decline of specific penicillin production rate—100% (depending on inducer and culture conditions)	Formation of white fluffy mycelial sectors, decreased spore pigmentation, (no) reduced conidiation	Christensen et al. 1995; Clutterbuck, Lovell and Raistrick, 1932; Douma et al. 2011; Künkel et al. 1992; Righelatoa 1976
	Adipoyl-7 aminodeacetoxycephalosporanic acid production	100%	White colonies	Robin et al. 2003
*Trichoderma reesei*	Cellulase production	10–100% (depending on the strain)	No information	Martzy et al. 2021

### Degeneration of penicillin production in *Penicillium chrysogenum*

Since its coincidental discovery in 1928, penicillin has been the oldest and is still considered the most-used antibiotic. Natural penicillin is produced by the filamentous fungus *Penicillium chrysogenum* (Fleming 1929; Dumancas et al. 2014). Already in 1932, the loss of penicillin productivity in *P. chrysogenum* was reported (Clutterbuck et al. 1932). Clutterbuck and co-workers noticed that the reduction of penicillin and chrysogenin after subculturing accompanied a change in the mycelium, which became less green and formed white flaky patches. Over time penicillin production yield and rate of *P. chrysogenum* increased manifold through random mutagenesis and selection rounds (Newbert et al. 1997; Miguel A. Peñalva et al. 1998). However, the loss of penicillin productivity was also observed in further evolved strains during extended carbon-limited chemostat fermentations (Righelatoa 1976; Künkel et al. 1992; Christensen et al. 1995; Vangulik et al. 2000; Douma et al. 2010). The application of batch or fed-batch fermentation for large-scale production of antibiotics is economically unfavorable due to a higher downtime and, thus, inefficient use of equipment (Douma et al. 2011). The degeneration of product formation in *P. chrysogenum* was also found for another antibiotic, adipoyl-7 aminodeacetoxycephalosporanic (ad-7-ADCA) acid. In extended cultivation, another phenotype, which increased during cultivation in the chemostat and was referred to as white colonies, was observed (Robin et al. 2003).

The decline in penicillin productivity in *P. chrysogenum* was found to be connected to growth limitation triggered with a carbon source. No degeneration occurred if the growth-limiting substrate was ammonia, phosphate, or sulfate (Righelatoa 1976). The type of carbon source affected the severity of degeneration since degeneration was observed to be more pronounced with ethanol than with glucose or sucrose (Righelatoa 1976; Christensen et al. 1995; Douma et al. 2011). The observations of the morphological manifestation accompanied by the degeneration of penicillin production varies from a fluffy-white sector formation of the mycelium to a decline in spore pigmentation or only to an overall reduced conidiation (Clutterbuck, Lovell, and Raistrick 1932; Righelatoa 1976; Christensen et al. 1995). Christensen and co-workers studied the stability of high-yielding strains obtained from Novo Nordisk A/S in continuous cultivation under glucose limitation. Two mutants with impaired penicillin productivity were isolated, which showed decreased spore pigmentation. The loss of penicillin productivity was traced back to genetic events leading to a different copy number or the loss of the genes of three key enzymes of the penicillin synthesizing pathway. The non-producing mutant was found to have a growth advantage over the producing type, reaching higher biomass yields (Christensen et al. 1995). In another study, penicillin production decline in aneuploid strains was associated with genetic instability and segregation into near wild-type phenotypes with faster growth, higher sporulation, UV sensitivity, and a decrease in DNA content, conidial, and nucleolus size (Künkel et al. 1992).

In contrast to the studies mentioned above, the most recent study on this topic on the degenerated version of an industrial *P. chrysogenum* strain from the DSM Biotechnology Center did not show any change in the gene copy number of the penicillin gene cluster. A rapid more than tenfold decrease in penicillin production occurred in prolonged chemostat fermentation with ethanol as a carbon source. The fact that this happened within 12 generations (500 h) argues against random mutagenesis as a possible reason. The levels of the first two enzymes, δ-aminoadipyl-L-a-cystenyl-D-a-valine synthetase (ACVS) and Isopenicillin-N synthase (IPNS), of the penicillin biosynthetic pathway were found to be reduced significantly. A transcript analysis revealed no significant changes in transcript levels of genes encoding critical enzymes or global regulators of secondary metabolism, which are involved in penicillin biosynthesis. However, a strong downregulation of sulfur and nitrogen metabolism and an intracellular decrease of the ß-lactam precursors valine and alpha-aminoadipate acid levels were observed. This was interpreted as more likely to be an outcome of the reduced need for penicillin precursor rather than the cause of the degeneration. The heterogeneity of the culture was presumed as one possible explanation for the effect of the strain degeneration phenomenon observed in this study. A theoretical simulation of a non-producing cell type outcompeting the productive type showed that 15% of the population would have to consist of the non-penicillin-producing cell type to explain the observations (Douma et al. 2011). However, as several other authors described, no different phenotype was identified in this study (Righelatoa 1976; Christensen et al. 1995). Another stated possible reason for this phenomenon is a substantial decrease in the translation efficiency of ACVS and IPNS during degeneration. The decline in penicillin productivity could only slightly be recovered by re-cultivation in unlimited batch culture and following chemostat culture with carbon limitation (Douma et al. 2011).

The characteristics of the degeneration of penicillin productivity are fundamentally different in differently evolved *P. chrysogenum* strains. Several distinct phenotypic changes were observed, indicating that there are multiple mechanisms contributing to the loss of penicillin production. To fully understand and prevent this phenomenon, further research is still required.

### Degeneration of cellulase production in *Trichoderma reesei*

Due to its extraordinary secretion capacity, *T. reesei* is exploited as a workhorse for industrial carbohydrate-active enzyme production. Cellulases of *T. reesei* are among the most abundant enzymes in the biotechnological industry and are applied for diverse applications such as bioethanol production, pulp, paper, food, or textile industry (Peterson and Nevalainen 2012). Degeneration of *T. reesei* is reported to occur spontaneously during the industrial production process leading to a loss of cellulase production capacity. The specific cellulase productivity of the industrial hypercellulase-producing *T. reesei* strain Iogen-M10 in a 14-L bioreactor started to decrease after 72 h and then plummeted to almost zero after 144 h. At the same time, an increase in a formation of a new non-cellulase-producing phenotype, the so-called (cel-) type, accompanied by a rise in biomass formation, was observed. The (cel-) type is defined as an isolate showing no clearance zone on an acid-swollen cellulose medium and was observed to outcompete the productive type and dominate the total cell population at the end of the cultivation. In the mid to late cultivation stages, an intermediate state, a so-called semi (cel-) type, was identified, gradually evolving to the full (cel-) type. This slow and gradual development to the non-productive type indicates that an epigenetic mechanism could cause degeneration. The loss of cellulase production was reported as irreversible since sub-cultivation of the (cel-) strains on fresh full or inducing medium could not restore cellulase productivity. Further, the degenerated strain had a reduced ability to metabolize several carbon sources such as lactose, cellobiose, xylose, ribose, and arabinose (Martzy et al. 2021).

Martzy and co-workers (2021) developed a protocol for small-scale, artificially induced strain degeneration that allows to obtain a percentage of (cel-) occurrence of the total cell population and calculate a degeneration rate for a strain and compare the degeneration behavior of differently evolved strains. The wild-type strain QM6a was compared to Rut-C30, which was derived from QM6a after three rounds of mutagenesis and screening for increased protein production, Iogen-M4, a moderate industrial cellulase producer, and Iogen-M10, a top producing strain, which all belong to the same strain lineage. No degeneration was found in QM6a, Rut-C30 degenerated to a small extent, followed by Iogen-M4, having a degeneration rate of approx. 10%, and Iogen-M10 degenerated to almost 100%. These results suggested that the severity of degeneration correlates with the productivity of a strain. It was considered as that secretory stress might influence the extent of degeneration. A mutation of Xyr1 (Xylanase regulator 1), the main transactivator of cellulase and xylanase expression, was suspected as an apparent cause for the degeneration. But no difference in the *xyr1* gene sequence was determined between producing and non-producing strains. However, the chromatin in promotor regions of cellulase-related genes was found to be more condensed in the degenerated Iogen-M10 than in the productive Iogen-M10, which is also reflected in the significantly decreased transcript levels of these genes. This observation also points towards an epigenetic mechanism as a potential cause. However, genetic mutations as a possible reason for the degeneration are not entirely ruled out yet (Martzy et al. 2021), and generally, the degeneration of cellulase productivity in *T. reesei* is not really understood. It requires further investigations to determine the underlying mechanisms that led to the observations described by Martzy and co-workers.

### Degeneration of citric acid production in *Aspergillus niger*

Citric acid is one of the world-wide most essential bulk chemicals and food additives, which is 99% produced by the industrial workhorse *A. niger*. More than 70% of total global production of citric acid (1.5 million tons annually) is produced in China (Xie et al. 2018; Behera 2020). Spontaneous degeneration of citric acid productivity has been reported for *A. niger* strain YX-1217, typically used for large-scale production in China. In 60 h of cultivation in a bioreactor at 38–39 °C using corn starch hydrolysate, this strain typically produces 180–200 g/L of citric acid (Hui et al. 2017). The reduction of citric acid production capacity in a degenerated strain is approximately 30–70% of the productive type. To maintain the productivity of the productive type, *A. niger* YX-1217 must be regularly rejuvenated (Xie et al. 2018). The spore morphology of the degenerated strain (YX-1217G) differs significantly from YX-1217. The spore surface of YX-1217G is smooth with larger holes instead of rough, showing many ridges.

Further, the colony color changed from black to dark brown, resulting from a thinner and partially lost melanin layer of the spore surface (Hui et al. 2017). The reduction of citric acid productivity is accompanied by increased biomass production, and interestingly, the degenerated strain produces a significantly higher amount of oxalic acid. The latter indicates that the degeneration also affects other organic acids (Hui et al. 2017; Xie et al. 2018). An extensive study by Xie and co-workers (2018) compared the transcriptomes of YX-121, YX-1217G, and the wild-type strain ATCC1015. In the exponential phase of citric acid production, genes related to the biosynthesis of citric acid, including the tricarboxylic acid cycle, glycolysis, starch and sucrose metabolism, and pyruvate metabolism, were downregulated in the degenerated compared to the productive strain and the wild-type strain.

Further, the degenerated strain showed a weaker hydrolase system for carbohydrate usage, a downregulation in genes coding for amino acid transporters, and a weaker transporter capacity. The central metabolism did not differ substantially (Xie et al. 2018). Currently, no explanation or cause of the degeneration of citric acid productivity and its underlying mechanisms in *A. niger* is available in the literature.

### Degeneration of hydrolytic enzyme production in *Aspergillus oryzae*

*A. oryzae* is widely applied in Asia to produce traditional fermented food such as soy sauce, rice wine, and soybean paste (Park et al. 2017). The ability of *A. oryzae* to secrete an enormous number and amount of hydrolytic enzymes is fundamental for the degradation of the biomass for subsequent fermentation and the formation of the characteristic soy sauce flavor (Ding et al. 2019). *A. oryzae* is used as a starter for the so-called koji fermentation, which consists of a 3-day solid state fermentation on a mixture of wheat flour and soybeans. This is the first step in producing soy sauce and is critical for the resulting flavor (Huang and Teng 2004; Gomi and Abe 2008). Strain degeneration of an industrial *A. oryzae* has been reported for the Chinese strain RD2 (Zhong et al. 2018). *A. oryzae* RD2 degenerates after repeated use, showing reduced productivity of hydrolytic enzymes and flavor precursor formation, resulting in a distasteful soy sauce. This was accompanied by a higher mycelium growth rate, reduced conidiation, and a formation of sectors with white hyphae and sclerotia formation, resulting from abnormal branching and intertwining mycelium (Jin et al. 2011; Zhong et al. 2018). Zhong and co-workers (2018) compared the whole genome and whole transcriptome of the productive (RD2) and the non-productive strain (TS2). The authors could explain the phenotypic degeneration by gene mutations related to the production of hydrolytic enzymes and flavor precursors. The underlying reason for the growth advantage of TS2 is described as an upregulation of amino acid catabolism and central carbon metabolism (Zhong et al. 2018). Mutants of another frequently used Chinese *A. oryzae* strain 3.042 are reported to show genetic instabilities (Ding et al. 2019).

## Possible mechanisms of strain degeneration

**Fig. 1:**
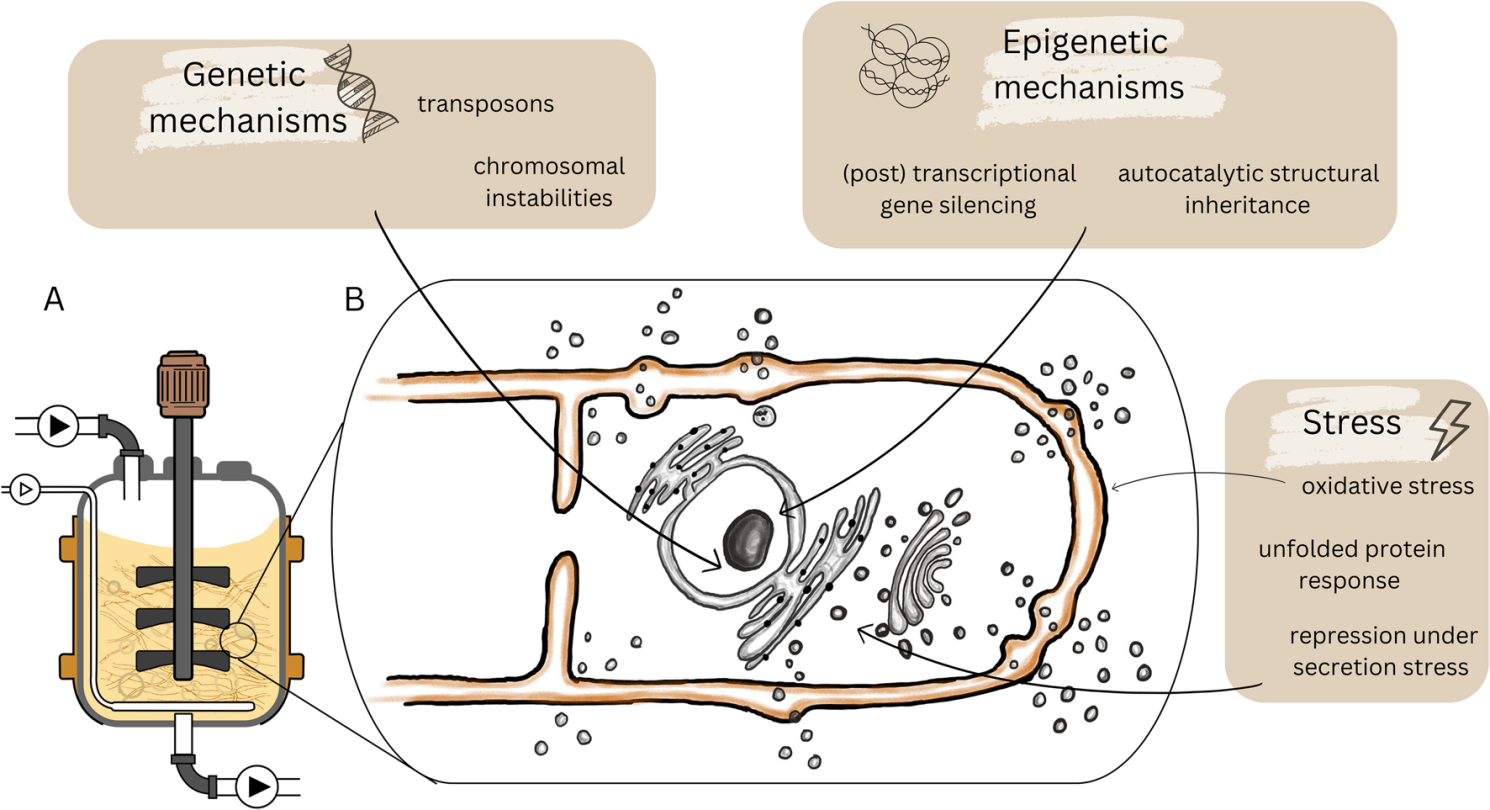
Overview on mechanisms involved in the phenomenon of fungal degeneration in a biotechnological process. (**A**) Schematic depiction of a bioreactor cultivating a filamentous fungus. (**B**) Enlarged section showing a hyphal tip including the nucleus and the secretory pathway. The boxes summarize the genetic, epigenetic, or stress-related mechanisms found to may be involved in fungal degeneration during a biotechnological process. Black arrows indicate where mechanisms possibly occur within the fungal hyphae

Poor performance in bioprocesses due to phenotypic variation can be divided into two scenarios. A temporal phenotypic variation describes a subpopulation of non-producing cells that temporarily ceases production, but then, at an unpredictable time point, production is recommenced. The other possibility is the irreversible loss of production capacity from a subpopulation during the fermentation. Due to the high biosynthetic activity requirement of the cells in an economically feasible bioprocess, the non-producing population may have a selective advantage in an industrially relevant timescale. A sum of effects leads to this selective advantage of the non-producer cells over the producing cell type. Enzyme synthesis, protein misfolding, DNA synthesis, accumulation of toxic byproducts, and losses of endogenous substances pose a high metabolic burden of extensive production and result in a pathway-specific fitness cost (Avery 2006; Carlquist et al. 2012; Rugbjerg et al. 2018). The literature that describes examples of degeneration in biotechnologically relevant fungi largely lacks explanations of the underlying mechanisms causing the observed phenotypic and biochemical changes. Generally, strain instability in fungi can be caused by genetic mechanisms in the nucleolus or the mitochondria or epigenetic phenomena affecting nucleic acid levels (RNA or DNA), proteins, or regulatory networks (Butt et al. 2007; Silar 2019). Genetic changes due to mutations or other genetic mechanisms can arise spontaneously even in relatively stable environments. These are often associated with a higher growth rate since the new trait can provide a growth or survival advantage for the specific environment. In contrast to this, epigenetic changes are highly influenced by the environment, as they are often triggered by external factors such as temperature, pH, or nutrient availability. Epigenetic changes can be more complex and require a higher investment of cellular resources and energy than genetic mechanisms. Due to this diversion of resources, the appearance of epigenetic mechanisms often is associated with a slower cellular growth rate (Silar 2019). However, several processes might even be active simultaneously; therefore, in many cases, it is impossible to identify an isolated mechanism as a causative for fungal degeneration (Reusser 1963). Other influential factors for strain degeneration are culture conditions or stress (Kreiner et al. 2002; Pitchaimani and Maheshwari 2003; Li et al. 2008, 2014; Pérez et al. 2021). The following chapters will briefly elaborate on the potential underlying mechanisms causing fungal degeneration in general and connections to the loss of productivity in biotechnologically relevant fungi will be made where possible. A summary of the likely reasons and mechanisms underlying the degeneration of fungi in a biotechnical process is depicted in Fig. 1.

### Genetic mechanisms

Random genetic mutations are a natural process and occur at a low frequency as a driving force of evolution. A high frequency of mutations can result from two scenarios (Silar 2019). A high mutation rate in fungi can be for example related to the activity of transposable elements or chromosomal instabilities, such as ploidy changes or chromosomal rearrangements (Daboussi 1996; Forche et al. 2018). Mutants resulting from a low mutation rate may arise at a high frequency due to positive selection. The new phenotype has a selective growth advantage and usually takes over the wild-type (Silar 2019). The latter was observed in most of the examples of fungal degeneration in a biotechnological process, such as by Zhong and co-workers (2018) on degeneration of hydrolytic enzyme productivity by *A. oryzae*, or by Christensen and co-workers (1995) on decline in penicillin productivity by *P. chrysogenum*. In one case of degeneration of penicillin production capacity in *P. chrysogenum*, a genetic mechanism involving chromosome instability was a postulated cause for the loss of penicillin biosynthetic genes. High penicillin-producing strains contain a higher copy number of the biosynthetic gene clusters as tandem repeats (Barredo et al. 1989; Smith et al. 1989; Fierro et al. 1995; Newbert et al. 1997). These amplifications are suspected to be a result of chromatid misalignment and recombination assisted by recombinogenic regions. The exact mechanism could also induce the deletion of penicillin biosynthesis-related genes (Newbert et al. 1997). The presence of multiple penicillin cluster genes, especially in extended cultivations under unfavorable conditions for penicillin production, may be a disadvantage for the fungus. In these conditions, selection pressure is given to cells without multiple penicillin cluster genes (Douma et al. 2011).

Another point to consider for the above-mentioned occurrence of genetic mechanisms related to the degeneration phenomenon is the genetic background of the respective fungal production strain. The past hyper-productive strains of filamentous fungi were obtained mainly by random mutagenesis. Recent efforts in genetic engineering enabled to even further improve production strains in particular through the application of CRISPR/Cas9 gene-mediated editing (Wang et al. 2020). The inactivation of the non-homologous-end-joining (NHEJ) pathway improved the efficiency of homologous recombination and is often applied in combination with CRISPR/Cas9 (Carvalho et al. 2010). However, several groups have reported that a deletion of this system can result in an increased vulnerability to DNA-damaging conditions and reduces cellular fitness. Therefore, NHEJ-deficient strains might be more prone to mutagenesis. Generally, it is recommended to only transiently disrupt the NHEJ pathway and restore it after transformation in order to avoid an unstable strain (Nielsen et al. 2008; Yang Chum et al. 2017).

### Epigenetic mechanisms

The epigenetic mechanisms in fungi regulating gene expression on the nucleic acid level are transcriptional and post-transcriptional gene silencing or activation. Transcriptional gene silencing is linked to DNA methylation and condensation of the chromatin structure and is reported to cause phenotypic diversity and instability through position effect variegation especially (Gottschling et al. 1990; Silar 2019). Post-transcriptional gene silencing, associated with interfering non-coding (nc) RNAs, was shown to be responsible for phenotypic switches in fungi (Nicolás and Garre 2016). Actual demonstrations of epigenetic mechanisms on nucleic acid levels involved in fungal degeneration are relatively rare. The degeneration of virulence in *Conidiobolus obscurus* was found to be potentially related to long nc (lnc) RNAs (Ye et al. 2021). The lnc RNAs identified in the study by Ye and co-workers target genes coding for heat shock proteins, secretory proteins, autophagy proteins, transporters, and other proteins related to stress response, potentially reducing virulence (Ye et al. 2021). Also, the degeneration of cellulase productivity in *T. reesei* was hypothesized to be rather caused by an epigenetic mechanism. Chromatin was found to be more condensed in promoters of cellulase-related genes in the degenerated population compared to the producing population (Martzy et al. 2021).

Another example of epigenetic gene silencing by heterochromatin is the observed occurrence of unstable phenotypes of the fission yeast *Schizosaccharomyces pombe*. It develops stable and unstable epimutations depending on their heterochromatin status, resistant to caffeine when grown with low caffeine concentration. Unstable isolates harbor noticeable heterochromatin islands with the respective declined expression of embedded genes related to caffeine resistance. These also show cross-resistance to other antifungal agents. Depending on the environmental conditions, heterochromatin-related processes may influence fungal phenotypes (Torres-Garcia et al. 2020). Finally, in budding yeast, phenotypic heterogeneity was reported to be mediated by introns, which results in a fitness advantage for the yeast coping better with starvation. Introns can induce phenotypic heterogeneity in response to environmental conditions (Lukačišin et al. 2022).

The protein-based epigenetic mechanisms are subdivided into the structural inheritance, created by autocatalytic structural alterations of macromolecules, and the topologic inheritance, relying on the topology of regulatory networks. Specially prions and other autocatalytic structural modifications of proteins might result in fungal phenotypic instability (Silar 2019). These can spread information embedded in their conformation through inheritance to the next generation, and in some cases, it can also be transmitted to other species. Prion formation usually leads to a non-functional protein, which is trapped in amyloid aggregates and cannot perform its cellular function (Graziani, Silar, and Daboussi 2004; Daskalov et al. 2012; Watanabe et al. 2018). Generally, not many prions or prion-like elements have yet been discovered in filamentous fungi so far. One example is the *σ* autocatalytic structure causes the Secteur phenotype in *Nectria haematococca* (Graziani et al. 2004). In yeasts, the *S. cerevisiae* prion [PSI +] was shown to be involved in forming several new unique phenotypes (True and Lindquist 2000). Also, the formation of the prion-like element [GAR +] in *S. cerevisiae*, which is supported by bacteria, leads to the formation of a new phenotype during the first step in sake production (Watanabe et al. 2018). Notably, there is a lack of reports on the degeneration of productivity due to autocatalytic structures. However, it cannot be excluded that protein-based epigenetic mechanisms could lead to culture degeneration in a biotechnical process.

### Stress mechanisms and fungal degeneration

The exploitation of fungi for hyperproduction in a biotechnological process subjects the fungus to an increased stress level. Some authors demonstrated that the observed change in culture morphology, metabolism, and product synthesis of fungi occurs in response to a stressor. Generally, productivity is closely related to fungal morphology, especially in filamentous fungi (Cox et al. 1998; Schügerl et al. 1998; Kreiner et al. 2002). The product formation depends on the macromorphology, which is between either pelleted or free filamentous form. The differentiation of the fungal hyphae is assumed to be highly important (Kreiner et al. 2002). The secretion of proteins is mainly associated with the apical tip region, and secondary metabolite secretion, such as penicillin, is assumed in the more mature hyphae regions (Peberdy 1994; Paul and Thomas 2000). Some authors even suggested that fungi change their morphology specifically to decrease stress (Hansberg and Aguirre 1990; Duran et al. 2010). For these reasons, it is unsurprising that a change in fungal morphology often accompanies the loss of production capacity in a biotechnological process.

In many cases of fungal degeneration or phenotypic instabilities, stress mechanisms are either directly associated with the occurrence of the phenomenon or have an amplifying effect on the severity. There are different stress mechanisms related to the degeneration of fungi, such as oxidative stress, unfolded protein response, and the repression of genes encoding secreted proteins in response to secretion stress (Sakekar et al. 2021). The accumulation of reactive oxygen species is linked to mitochondrial dysfunction and the deformation of fungal mycelium of *Metarhizium anisopliae*, as well as *A. nidulans* (Li et al. 2008, 2014). The phenomenon of cellulase productivity degeneration in *T. reesei* was found to be more pronounced the further evolved the strain is, indicating a connection between the productivity of the strain, the degeneration severity, and endoplasmic reticulum stress (Martzy et al. 2021). At times of high protein production, *T. reesei* responds to stress caused by the accumulation of unfolded protein in the endoplasmic reticulum with a feedback mechanism to reduce the transcript levels of the respective genes (Pakula et al. 2003). This feedback mechanism was proven to exist in *A. niger* and was found to downregulate glucoamylase expression selectively under endoplasmic reticulum stress. This exact mechanism is speculated to contribute to the degeneration phenomenon of citric acid productivity by downregulating genes involved in citric acid production (Al-Sheikh et al. 2004; Xie et al. 2018).

Furthermore, genome instability due to extensive chromosomal modifications in fungi is shown to arise more frequently under stress conditions (Forche et al. 2018). The intron-related phenotypic instability of the budding yeast is induced by osmotic stress. This mechanism is seen as a stress-survival tool of this yeast to adapt to continued stress conditions (Lukačišin et al. 2022). Many lnc RNAs causing virulence degeneration in *Conidiobolus obscurus* are related to the response of the fungus to environmental stress (Ye et al. 2021).

## Prevention or circumvention of fungal degeneration

The progeny of cells is subjected to genetic variation during growth and reproduction as a result of evolution. The stability of a strain can be maintained by suppressing mutational mechanisms or by constant selection for the desired phenotype. Another important measure is adequate storage and preservation of the respective microorganism. However, the degeneration of fungal productivity can also occur spontaneously during a production process, which directly leads to economic losses. The best approach how to prevent such degeneration might depend on the fungal strain, the target product, and the particular process. Therefore, it is essential to determine the characteristics of the specific phenomenon and to understand the underlying mechanisms to identify promising targets for prevention. On the other hand, to fundamentally avoid degeneration in a biotechnological process, the fungal strain should be selected for stability and the bioprocess designed accordingly. The following chapters will briefly summarize the methods commonly applied for fungal culture preservation and the characterization of strain degeneration in biotechnologically relevant fungi and discuss suggested approaches from the literature on designing a stable bioprocess using fungi.

### Preservation of fungal cultures

Storage methods of fungal cultures have to ensure genetic and morphologic stability of the strain besides sustaining long-term viability. Generally, the methods can be subdivided into preservation at temperatures above freezing, by freeze-drying, and at a frozen state. For a short period, fungal cultures can be kept at temperatures above freezing on nutrient agar plates at room temperature or + 4 °C. Periodic inspection on morphological changes and/or bioassays is recommended, especially after serial transfer because it can induce morphological and physiological changes. It was reported that microbial culture slants under mineral oil or distilled water can be kept viable for decades (Humber 1997; Nakasone et al. 2004). While the relation between storage and the fungal productivity are not fully known, it should be noted that a decline of sporulation capacity and the loss of virulence was reported for strains kept with mineral oil (Mendes Da Silva et al. 1994; Al-Bedak et al. 2019). The most widely used long-term storage is cryopreservation by freezing at − 20 °C or − 80 °C with the help of cryoprotectants or at − 196 °C using liquid nitrogen. Long-term preservation by lyophilization is not applicable for all fungi; however, it can maintain cultures at a low cost for up to 30 years. Some practical and effective preservation methods for specific types of fungi use various organic substrates, such as straw, plant or insect tissue, filter paper, wood chips, soil, or sand (Nakasone et al. 2004). A recently developed low-cost method for the long-term storage of filamentous fungi uses cotton balls moisturized with potato dextrose agar. No morphological change was observed for up to three years in 135 fungal strains (Al-Bedak et al. 2019).

### Strain selection and bioprocess design

The development and selection of fungal strains is the first step in bioprocess design and usually focuses on identifying the highest-producing strain. However, to avoid spontaneous culture degeneration and production losses, these initial steps should also consider critical factors such as morphological characteristics, strain stability, and stress tolerance to ensure robust and reproducible large-scale production.

As discussed above, several studies have pointed out the importance of fungal morphology on product formation and secretion. Fungal morphology in submerged fermentations, which is the most applied cultivation process for filamentous fungi, can exhibit various forms such as differently branched disbursed hyphae, mycelial clumps, or compact pellets. Up to now, the development of morphology is often unpredictable and uncontrollable (Sakekar et al. 2021; Lübeck and Lübeck 2022). Generally, morphology relates to the specific strain and is influenced by culture conditions, including inoculum quality and concentration, media composition, and mechanical forces. Rheology depends on fungal morphology, highlighting the link between fungal productivity and morphology (Posch et al. 2013). Morphology analysis and control are required for a stable bioprocess to understand the fungal morphological response to a specific condition. Recently developed techniques to create a specific desired fungal morphology are summarized under the term “morphology engineering.” These efforts are based on genetic, micro-, and macroparticle and salt-enhanced methods (Meyer et al. 2021). The additions of microparticles, such as talc or aluminum oxide, affect the morphology in a strain-specific manner. Although they were proven beneficial for tailor-made morphology, the mechanisms behind them are not yet fully understood. Using titanate microparticles with *A. niger* led to pellets with a dense microparticle core. The salt-enhanced cultivation is based on increasing osmolarity by adding salts leading to more mycelial and clump growth. Morphology can be manipulated in a desired way by adding different ions, thereby influencing productivity (Böl et al. 2021). Furthermore, recent “omics” studies have discovered genes related to optimized morphology, secretion, and productivity. This knowledge enables the application of genome editing approaches to tailor a specific morphology for optimal production (Sakekar et al. 2021). To understand and predict the fungal response to process parameters, diverse morphological parameters could be characterized in a high-throughput approach, including morphological classification and frequency distribution. Morphological classification involves the categorization of fungi based on their physical characteristics, while frequency distribution refers to analyzing how often these traits occur within a population. These methods provide knowledge on how diverse morphological characteristics influence the response of fungi to process parameters and the overall production performance. This further allows the development of a model based on population balancing to better understand heterogeneities affecting production performance (Posch et al. 2013).

The initial screening for the optimal candidate strain should include strain stability studies. The stability of a strain is usually tested by repeated transfer on a selective medium for several generations while monitoring growth and productivity and fungal morphology. The inoculum type for the stability tests can vary from single-spore or multi-spore to mycelial transfer and influences the observed effects. The medium used for the subculturing and the applied methods for product analysis affect the results (Shah et al. 2007). The number of transfers should be aligned with the expected number of generations in the bioprocess. Considering an industrial bioreactor with a size of 200 m^3^, approximately 60 to 80 generations are required to reach a population size of 10^20^ cells starting from a master cell bank aliquot (Rugbjerg et al. 2018). Generally, it is recommended to closely relate the characteristics of the stability screening to the bioprocess conditions to predict the strain stability after scale-up accurately.

The close interconnection between fungal morphology, productivity, and stress suggests the importance of studying the response of a strain to deal with different stressors relevant to the bioprocess. The primary stress responses of interest for a fungal bioprocess are—as mentioned earlier—response to oxidative stress, unfolded proteins, and secretion stress. Oxidative stress occurs during the fungal bioprocesses due to difficulties in oxygen transfer, creating dissolved oxygen tension (Kreiner et al. 2000). Reactive oxygen species can lead to the oxidation of nucleic acids or proteins, causing significant cell damage. Fungi contain a defense system against oxidative stress, capable of disposing of these reactive oxygen species to a certain extent. The antioxidant defense system in filamentous fungi is relatively understudied compared to bacteria or yeast (Halliwell 2006). Oxidative stress is investigated by exposing cells to significant quantities of oxidizing or superoxide radical generating substances, such as hydrogen peroxide, or more relevant for bioprocess using elevated dissolved oxygen tension. The parameters examined are usually the activities of oxidative defensive enzymes. Culture morphology changes should be analyzed as well as described above (Kreiner et al. 2002). An unnaturally high production of proteins results in the need for a higher capacity of the organism to fold and transport proteins correctly. The unfolded protein response as well as the repression of genes encoding secreted proteins in response to secretion stress are two known mechanisms occurring in filamentous fungi to overcome the harmful effects of the limitations of the secretory pathway. Typically, the response of cellular stress resulting from protein secretion is analyzed by treating the cells with a chemical agent that interferes with protein folding or transport. The response of *A. niger* and *T. reesei* to impaired protein folding was tested with dithiothreitol for both and additionally brefeldin A and the Ca^2+^ ionophore A23187 for *T. reesei* (Pakula et al. 2003; Al-Sheikh et al. 2004).

### Characterization of fungal degeneration

As described in the previous chapters, the phenomena of fungal degeneration are very diverse; thus, there is no universal solution to prevent it. The only way to understand a particular phenomenon and to find a tailor-made solution for its prevention is to investigate and elucidate the respective mechanism causing it. Generally, studying such a mechanism follows a fundamental principle as discussed below.

First, the respective fungus is either subjected to a laboratory scale, often extended incubation, which reflects as close as possible the production conditions or it is directly examined during the biotechnological production process. The first approach has the advantage that it can be replicated and, therefore, also used to monitor any measures for preventing strain degeneration. The development of such a replicable, laboratory scale degeneration protocol involves the identification of the conditions that strongly induce the synthesis of the target compound. For studying the penicillin degeneration in *P. chrysogenum*, different cultivation times and carbon sources such as glucose, sucrose, and ethanol were examined (Righelatoa 1976; Christensen et al. 1995; Douma et al. 2011). To investigate the cause of loss of cellulase productivity of industrial *T. reesei* strains, a laboratory scale protocol was developed that creates artificially degenerated strains comparable to the ones isolated during the production process. This protocol consists of a 120-h-long incubation in cellulase-inducing conditions followed by a plate screening to select between cellulase-producing and non-producing cell types (Martzy et al. 2021).

The second step of the characterization of fungal degeneration is the identification and isolation of degenerated cell types. This is usually done by an agar plate screening to select for a morphological or physiological different phenotype. When applied in a time course during the above-mentioned step, it will also identify the time point at which the productive strain starts degenerating. *P. chrysogenum* cell types impaired to produce penicillin were identified by their distinct appearance on agar plates after spore isolation compared to the morphology of the productive type (Christensen et al. 1995). In the case of degeneration of cellulase productivity in *T. reesei*, the screening was done by incubating spores on carboxymethylcellulose agar plates and subsequent staining with Congo red to observe clearance zones. Clearance zones depict the ability of the colonies to produce cellulase (Martzy et al. 2021). Regarding the degeneration of citric acid production in *A. niger* and the degeneration of *A. oryzae*, such a screening is not described in the literature. The degenerated phenotypes of these strains were isolated at the end of the industrial bioprocess (Zhong et al. 2018; Xie et al. 2018).

The third step is the comparison of the degenerated phenotype to the productive phenotype regarding morphological, physiological, metabolic, and genetic, or epigenetic changes. Identifying the differences might allow to trace them back to the mechanism causing the phenomenon. If the metabolic pathway of the product is known, the quantification of protein levels of pathway enzymes and a flux balance analysis could be implemented to see if there are metabolic changes (Douma et al. 2011). Whole-genome comparison can identify whether the DNA sequence of the degenerated strain differs from the productive strain. Subsequent analysis of the potential mutation sites can suggest whether they are a result of a DNA mutation event by genetic or epigenetic origin. The additional application of a whole transcriptome comparison might identify key players involved in the degeneration phenomenon and reveal promising targets for further investigation (Zhong et al. 2018). Further, potentially identified key players of degeneration could be genetically modified by gene editing tools to specifically prevent strain degeneration. Whole transcriptome studies investigating the degeneration phenomena of *A. niger*, *A. oryzae*, and *P. chrysogenum* further identified potential target sites to enhance the production yield (Douma et al. 2011; Zhong et al. 2018; Xie et al. 2018). In case there is evidence for epigenetic regulation underlying the degeneration phenomenon, further analysis of DNA methylation, chromatin status, and histone modifications should be aimed for.

Another interesting approach to characterize fungal degeneration in a biotechnical production process is applying a model to explain the dynamics in a heterogeneous population in the bioreactor (Flevaris and Chatzidoukas 2021). Rugbjerg and co-workers developed a model to quantify the enrichment of spontaneous mutants while simultaneously using ultra-deep time-lapse sequencing to identify genetic errors (Rugbjerg et al. 2018, 2021). The application of such a model helps to predict the effect of the degeneration on the bioprocess.

## Concluding remarks

Very little is known about the reasons that cause fungal cultures in the laboratory or a biotechnological process to degenerate, and research lacks to identify the underlying mechanisms. Evidence of a loss or attenuation is present for diverse product types, including industry-leading products. Observed strain degeneration phenomenon ranges through the most well-known and most used fungi in biotechnological production, such as *P. chrysogenum*, *T. reesei*, and *A. niger*. Even though the types of products and strains affected by degeneration seem random, the cases show a similar pattern. Degeneration leads to a new fungal phenotype, often with different morphological and physiological attributes and impaired productivity upon serial subculturing or in a spontaneous event within the biotechnological process. Simultaneously, this phenomenon frequently is accompanied by increased biomass formation. It is apparent that if the fungi lose production capacity, more energy and substrate can be used for growth, serving as a competitive advantage to the non-producing cells. The origin of this phenomenon can be found in genetic or epigenetic, as well as stress-related mechanisms, and differs in each case. Overcoming the current limitations of fungal bioproduction is highly important to enable the necessary transition to a more sustainable industry. In this respect, research is required to conquer the phenomenon of degeneration in industrial-relevant fungi, ideally by tailored measures as soon as the causal mechanism(s) are identified and understood.
